# Efficacy of an Experimental Occlusion Technology Toothpaste in the Relief of Dentinal Hypersensitivity: An 8-week Randomised Controlled Trial

**DOI:** 10.3290/j.ohpd.b1075109

**Published:** 2021-03-17

**Authors:** Jonathan E. Creeth, Gary R. Burnett

**Affiliations:** a Principal Scientist, Medical Affairs, GSK Consumer Healthcare, Weybridge, Surrey, UK. Experimental interpretation, wrote manuscript, approved final manuscript.; b Principal Scientist, Clinical Research, GSK Consumer Healthcare, Weybridge, Surrey, UK. Experimental design and interpretation, wrote manuscript, approved final manuscript.

**Keywords:** dentifrices, dentin hypersensitivity, randomised controlled trial, tin fluorides

## Abstract

**Purpose:**

A novel anhydrous toothpaste formulation has been developed containing the anti-dentinal hypersensitivity (DH) ingredient stannous fluoride (SnF_2_).

**Materials and Methods:**

This randomised, controlled, examiner-blind, parallel-group, stratified (by baseline Schiff sensitivity score) study compared efficacy of an experimental ‘Test’ toothpaste (n = 67) containing 0.454% SnF_2_, 0.072% sodium fluoride and 5% sodium tripolyphosphate (all percentages w/w) with a negative ‘Control’ 0.76% sodium monofluorophosphate toothpaste (n = 68) in relieving DH in healthy Chinese adults. After 4–6 weeks acclimatisation, DH was assessed at baseline and following 4 and 8 weeks’ twice-daily brushing by response to evaporative (air) (Schiff sensitivity score) and tactile (Yeaple probe) stimuli. An analysis of covariance model was used (factor: treatment group; covariate: baseline Schiff sensitivity score).

**Results:**

Both Test and Control toothpastes statistically significantly reduced Schiff sensitivity score from baseline after 8 weeks’ use; the Test toothpaste also statistically significantly reduced the score after 4 weeks’ use (all p < 0.001). The Test toothpaste reduction was statistically significantly superior to the Control toothpaste reduction at both timepoints (p < 0.001). Percentage differences in treatment effects between Test and Control groups were 24.1% at 4 weeks and 31.7% at 8 weeks. Tactile threshold scores for both treatments statistically significantly increased from baseline at both timepoints (all p < 0.001); however, there were no statistically significant differences between Test and Control groups. Both toothpastes were well-tolerated with no adverse events reported.

**Conclusion:**

The Test toothpaste containing 0.454% SnF_2_ reduced DH statistically significantly more than the Control as evaluated by the Schiff sensitivity score, but not by tactile threshold.

Dentinal hypersensitivity (DH) occurs following a thermal, chemical, osmotic, or tactile stimulus.^5^ DH can arise when fluid-filled dentin tubules become exposed at the tooth surface, providing access to the live pulp. When a stimulus is applied to exposed dentin, fluid movement within dentinal tubules is postulated to trigger intra-dental nerves to transmit a pain impulse.^4^

There are currently two recognised approaches for DH relief consisting of depolarising or tubule-occluding agents.^1,37^ The latter physically block the top of dentin tubules, reducing dentin fluid movement in response to external stimuli.^29^ Occlusion-based agents may provide more rapid, sometimes immediate,^13-15,21,28^ DH relief than nerve depolarisation treatments.^2,24^

Stannous fluoride (SnF_2_) has long been used in DH relief products^34^ with the clinical efficacy of SnF_2_-containing toothpastes clearly demonstrated.^3,20,37^ The mechanism of action is believed to be via binding of cationic stannous ions to anionic sites on dentin surfaces, leading to precipitation of stannous and stannic oxides and hydroxides, together with salivary proteins and toothpaste-derived solids onto the dentin surface. These can occlude dentinal tubules.^9,22^

Regarding formulation of SnF_2_ toothpastes, there are several factors to consider. Stannous ions are susceptible to oxidation and hydrolysis in aqueous conditions.^22,31^ They have the potential to cause enamel surface staining due to stannous ions binding to the pellicle, but the mechanisms involved are not well understood.^10^ The SnF_2_ formulation used in this study has been developed to stabilise the stannous ions in an anhydrous base and to incorporate the stain-control agent sodium tripolyphosphate (STP), both of which dissolve and become fully active upon mixing with saliva.^20,35^ STP is a mild calcium-chelating agent that has an affinity for tooth surfaces, inhibiting attachment of stain molecules and facilitating their removal. This ingredient has been shown to reduce staining from stannous-containing toothpastes.^20,33^ Further, as polymers within a toothpaste can impact delivery of the stannous-based occluding layer to dentin, the polymer system of the experimental formulation was developed to optimise the speed and degree to which this occurs.^17^ Finally, the fluoride content has been increased from 1100 ppm provided by 0.454% (w/w) SnF_2_, to a total of 1426 ppm by addition of sodium fluoride. Clinical studies show that SnF_2_ formulations similar to that used here provide short-term (immediate to 14 days^7,8^) and long-term (8 weeks^26,27^) relief from DH.

Clinical data on the anhydrous SnF_2_ formulation have been obtained in North American and United Kingdom participants.^7,8,25,26^ The present study was conducted in China to establish effectiveness according to Chinese Ministry of Health guidelines. These state that for a treatment to be regarded as effective, a 15% difference in change from baseline between Test and Control toothpastes at 8 weeks should be demonstrated using two independent endpoints.^6^

As such, the objectives of this study were to compare clinical efficacy of an experimental 0.454% (w/w) SnF_2_ toothpaste in relieving DH, as measured by evaporative (air) sensitivity (primary objective) and tactile threshold (secondary objective), against that of a Control toothpaste, used twice-daily for 8 weeks.

## Materials and Methods

This was an 8-week, single-centre, controlled, randomised, examiner-blind, two-treatment, parallel-group, stratified (by maximum baseline Schiff sensitivity score of ‘test teeth’) study in healthy participants with self-reported and clinically diagnosed DH and at least two sensitive teeth. The study was conducted at a P.R. China-based university, was approved by an independent research ethics committee before initiation (State Drug Clinical Study Institution, Wuhan University Stomatological Hospital, Ethics Authorisation 06, 2016) and was conducted in accordance with the Declaration of Helsinki and local laws (clinicaltrials.gov: NCT02861664).

### Participants

Healthy adults aged 18–65 years with no clinically significant or relevant abnormalities on oral examination were recruited. Eligible participants were recruited through the clinical site’s existing database of people with DH who had a self-reported DH history of between 6 months and 10 years (verbally confirmed by the participant at the screening visit) and a minimum of 20 natural teeth, confirmed by examination at the screening visit. At screening, eligible participants had at least two accessible non-adjacent teeth with erosion, abrasion or facial/cervical gingival recession (EAR), a Modified Gingival Index (MGI) score of 0 adjacent to the test area, a clinical-mobility score ≤ 1 and a Schiff sensitivity score ≥ 1. At baseline, eligible participants had a minimum of two accessible non-adjacent teeth with signs of sensitivity as determined by a tactile stimulus threshold of ≤ 20 g and a Schiff sensitivity score ≥2.

Exclusion criteria included: pregnancy; breastfeeding; allergy/intolerance to study materials; a chronic debilitating disease; a condition/medication causing xerostomia; daily use of a medication/analgesic that could interfere with pain perception; current/recent use of antibiotics; requirement for antibiotic prophylaxis for dental procedures; mouth/tongue piercings; dental implants; gross periodontal disease; dental prophylaxis within 4 weeks, desensitising treatment or tooth bleaching within 8 weeks, scaling or root planing within 3 months, or treatment of periodontal disease within 12 months of screening. Exclusion criteria for Test teeth included: current/recent caries; treatment of decay within 12 months of screening; exposed dentin with deep, defective or facial restorations; abutments for partial dentures; full crowns or veneers, orthodontic bands or cracked enamel; sensitive teeth with contributing aetiologies other than EAR or not expected to respond to an over-the-counter anti-DH toothpaste. While it is acknowledged that some of these excluded Test teeth could be hypersensitive, they were excluded from analysis in this study either because the tooth condition meant the aetiology of DH was potentially complex and not necessarily due to exposed dentin tubules (e.g. due to caries or cracks) or that it might restrict accurate assessment of DH and/or restrict access of the sensitive site while brushing with the toothpaste (e.g. through the presence of orthodontic bands or partial dentures).

### Study Procedures

At the screening visit, participants provided written informed consent, and their demographic characteristics, medical history and medication use were recorded and an oral soft tissue (OST) examination was conducted. Dentition was assessed for evidence of EAR; gingival health status (MGI; score = 0);^19^ tooth mobility (modification of the Miller scale; score ≤1);^18^ and sensitivity to an evaporative (air) stimulus using the Schiff ensitivity scale (score ≥1).^32^ Eligible participants received a standard fluoride toothpaste (Colgate Strengthen Fresh, 1400 ppm fluoride as sodium monofluorophosphate; Colgate-Palmolive; Guangzhou, P.R. China) and toothbrush (Aquafresh Clean Control, GSK Consumer Healthcare [GSKCH]: Weybridge, UK) to use twice daily for 4–6 weeks between screening and baseline visits. First toothpaste use was carried out under study site supervision.

At the baseline visit, ongoing eligibility was assessed; any adverse events (AEs), incidents and medication changes were recorded, and acclimatisation toothpaste compliance was evaluated, based on participant-completed diaries. Following an OST examination, sensitivity of eligible teeth identified at screening was confirmed by response to a tactile stimulus administered by a Yeaple probe,^30^ which permits application of a controlled force to the dentin surface. Testing began at a pressure of 10 g and was increased by 10 g with each successive challenge. Following each challenge, participants were asked whether the stimulus caused pain or discomfort. The tactile threshold in grams was recorded when either the participant gave two consecutive ‘yes’ responses to the same pressure level or the maximum force (20 g at baseline, 80 g at post-baseline visits) was reached.

Five minutes after tactile stimulus evaluation, teeth with a tactile threshold of ≤20 g were evaluated for sensitivity to an evaporative (air) stimulus^32^ by directing a single 1-s jet from a dental air syringe from a distance of approximately 1 cm onto the exposed dentin surface of the isolated Test tooth. The examiner’s assessment of the participant’s response to the air stimulus was measured using the Schiff sensitivity scale: 0 = participant does not respond to air stimulus; or: participant responds to air stimulus and 1 = does not request discontinuation; 2 = requests discontinuation or moves from stimulus; or 3 = considers stimulus to be painful and requests discontinuation of the stimulus.^32^ The Schiff sensitivity assessment investigator selected two suitable, qualifying (Schiff sensitivity score ≥2), non-adjacent Test teeth for study evaluation. Two examiners performed the assessments throughout the study; one for tactile threshold, the other for evaporative (air) sensitivity.

Eligible participants were randomised to treatment according to a schedule provided by the biostatistics department of the study sponsor. Participants were stratified by maximum baseline Schiff sensitivity score (2 or 3). Randomisation numbers were assigned in ascending numerical order as participants were deemed eligible. The randomisation schedule was generated using an in-house validated program based on a random block design with a block size of four. The dental examiners, study statisticians, data management staff and other study-sponsor employees who could have influenced study outcomes were blinded to group allocation. For a study to be classed as truly double-blind, the toothpastes would have needed to have been identical in appearance, taste and texture and since this was not the case, this study is considered to be single-blind only.

Participants were randomised to the Test toothpaste containing 0.454% (w/w) SnF_2_ and 0.072% (w/w) sodium fluoride (1426 ppm fluoride total) or a negative Control toothpaste containing 0.76% (w/w) sodium monofluorophosphate as the sole source of fluoride (1400 ppm fluoride: Colgate Triple Protection, Colgate-Palmolive). Tubes were overwrapped to disguise identity. Participants brushed in their usual manner with a full ribbon of toothpaste for 1 timed min, twice a day (morning and evening) for 8 weeks, using the toothbrush provided at the start of the study. First use was under study-site supervision with a further supervised brushing at week 4.

Tooth sensitivity in response to tactile and evaporative stimuli was reassessed after 4 and 8 weeks. At each visit, participants underwent an OST examination and medication review. Compliance was assessed by review of participant-completed diaries.

Before study visits, participants refrained from oral hygiene procedures for at least 8 h, from eating/drinking for at least 4 h, and from excessive alcohol consumption for 24 h. Small sips of water were permitted within 4 h but not within 1 h of the visit. During the study, participants could not chew gum or use oral care products or any products to treat sensitive teeth other than those provided. Dental floss use was permitted for impacted food removal. Participants delayed any non-emergency dental treatment until they completed the study.

### Safety

Any OST examination abnormalities and spontaneously reported AEs were recorded from first brushing with acclimatisation toothpaste until 5 days after last use of study toothpaste. The investigator assessed the relationship between study toothpaste and AE occurrence using clinical judgement and graded the AE as mild (easily tolerated, causes minimal discomfort, doesn’t interfere with everyday activities), moderate (sufficiently discomforting to interfere with normal everyday activities) or severe (prevents normal everyday activities). Treatment-emergent AEs were reported for the safety population, which included all randomised participants who used study toothpaste.

### Data Analysis

To achieve approximately 60 participants completing treatment in each group, sufficient participants were screened so that approximately 180 entered the acclimatisation phase and approximately 130 could be randomised to treatment. The sample size was based on a computer-generated simulation program to meet Chinese Ministry of Health guidelines^6^ of 15% difference in change from baseline between Test and Control toothpastes at 8 weeks. It was estimated that 60 participants completing the study per group would provide >95% power to detect a difference of 0.4 in mean Schiff sensitivity score at 8 weeks, assuming a standard deviation (SD) of 0.578. A SD of 0.614 was actually observed, slightly greater than predicted from previous studies. Statistical analyses were conducted using SAS (version 9.4, SAS Institute; Cary, NC, USA).

Efficacy analyses were based on the modified intent-to-treat population, comprising participants who were randomised, received at least one dose of study treatment and provided at least one post-baseline assessment of efficacy.

The primary efficacy variable was Schiff sensitivity score change from baseline at week 8, calculated as participant-level Test-teeth mean. An analysis of covariance (ANCOVA) model was used (treatment group as factor; baseline Schiff sensitivity score as covariate). Adjusted mean change from baseline for each treatment and difference between treatments was calculated, together with 95% confidence intervals and p-values for between-treatment comparison. Week 4 Schiff data was analysed similarly. For tactile threshold (weeks 4/8), the ANCOVA model had treatment group and Schiff sensitivity strata as factors and baseline tactile threshold as covariate.

For all analyses, the assumption of normality was investigated and a non-parametric method (van Elteren test) was used if violated. The difference between treatment effects was calculated as percentage change in the Test product adjusted-mean value minus percentage change in the control adjusted-mean value.

## Results

**Fig 1:**
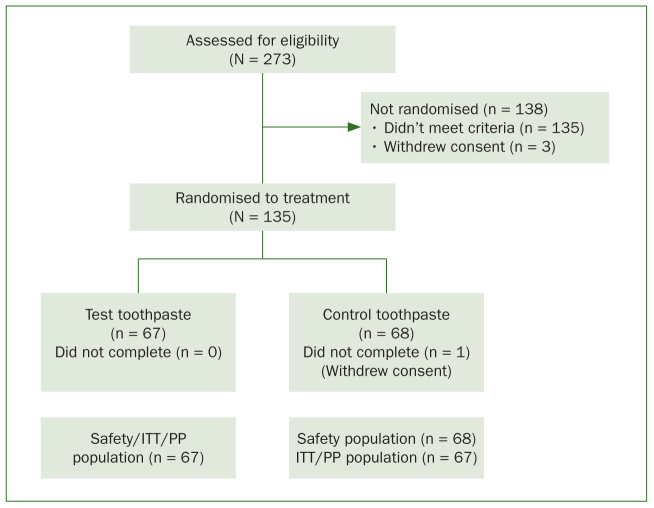
Participant disposition. mITT: modified intent-to-treat; N,n: number; PP: per protocol.

Of 273 screened participants, 135 were randomised to treatment (Fig 1). The first participant was enrolled on 21 September 2016; the last completed the study on 24 December 2016. Most participants were female (n = 122; 90.4%) and all were Asian; the mean age was 41.0 years (range 24–59 years). Demographic characteristics and baseline maximum Schiff sensitivity score stratification were similar between groups (Table 1). Overall, 47 participants (34.8%) had a maximum baseline Schiff score of 3 for the two selected Test teeth. The baseline scores between the groups were similar for both endpoints. Anonymised individual participant data and study documents can be requested for further research from www.clinicalstudydatarequest.com.

**Table 1:** Summary of baseline characteristics (safety population, N = 135)

Characteristic	Test toothpaste (n = 67)	Negative Control toothpaste (n = 68)
Sex, n (%)		
Male	5 (7.5)	8 (11.8)
Female	62 (92.5)	60 (88.2)
Age, years		
Mean (SD)	40.0 (8.21)	41.9 (8.78)
Range	24–58	24–59
Race, n (%)		
Asian	67 (100.0)	68 (100.0)
Maximum Schiff sensitivity score at baseline^a^, n (%)		
2	44 (65.7)	44 (64.7)
3	23 (34.3)	24 (35.3)

^a^Schiff sensitivity score of the two test teeth in response to evaporative (air) stimulus. n (%): number, percent; SD: standard deviation.

### Schiff Sensitivity Score

**Fig 2:**
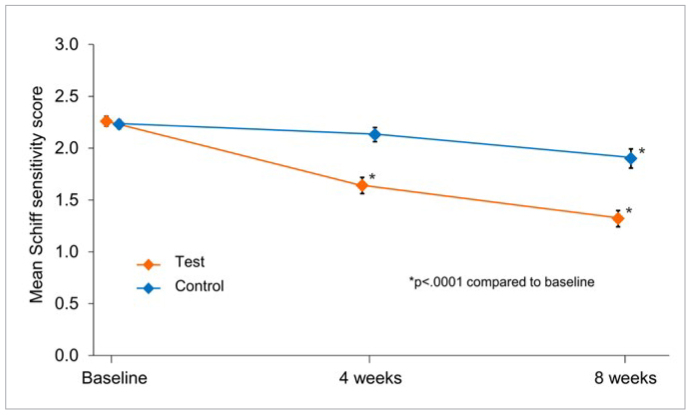
Schiff sensitivity score by treatment group and visit (modified intent-to-treat population). Data shown are raw means ± standard errors and are offset for clarity; a low score is favourable. Week 0 = baseline.

For the Schiff sensitivity score, the data were normally distributed and were analysed using ANCOVA methodology. There were statistically significant decreases from baseline for Schiff sensitivity score at weeks 4 and 8 for the Test toothpaste and at week 8 for the Control toothpaste (all p < 0.001) (Fig 2; Table 2). Compared with the Control toothpaste, there was a statistically significantly greater decrease in sensitivity from baseline at week 8 (p < 0.001) (primary endpoint) for the Test toothpaste group, with a 31.7% difference between groups. A statistically significant benefit was also observed at week 4 (p < 0.001), with a 24.1% difference between groups (Table 2).

**Table 2:** Adjusted mean changes from baseline in Schiff sensitivity score and tactile threshold and treatment comparison (modified intent-to-treat population, N = 134)

Timepoint	Test toothpaste (n = 67)	Negative Control toothpaste (n = 67)	Treatment comparison^a^	% difference in treatment effect^b^
**Schiff sensitivity score** ^a^				
Baseline^c^	2.26 (0.048)	2.23 (0.043)	–	–
Week 4^d^	-0.6 (-0.8, -0.5) p < 0.001 [-27.3%]	-0.1 (-0.2, 0.0) p = 0.0964 [4.2%]	-0.5 (-0.7, -0.3) p < 0.001	-24.1%
Week 8^d^	-0.9 (-1.1, -0.8) p < 0.001 [42.5%]	-0.3 (-0.5, -0.2) p < 0.001 [14.6%]	-0.6 (-0.8, -0.4) p < 0.001	-31.7%
**Tactile threshold (g)** ^a^				
Baseline^c^	11.1 (0.26)	10.9 (0.28)	–	–
Week 4^d^	4.8 (2.3, 7.4) p < 0.001 [43.4%]	4.8 (2.3, 7.4) p < 0.001 [47.4%]	-0.0 (-3.6, 3.6) p = 0.9968 p = 0.1903^e^	-0.0%
Week 8^d^	10.5 (6.7, 14.2) p < 0.001 [96.5%]	8.3 (4.6, 12.1) p < 0.001 [79.7%]	2.1 (-3.2, 7.5) p = 0.4334 p = 0.1945^e^	11.0%

^a^For Schiff sensitivity scores, a negative difference favours the Test toothpaste; for tactile threshold, a positive difference favours the Test toothpaste. ^b^Percentage difference between treatment effects, calculated as percentage change in adjusted mean for Test toothpaste minus percentage change in adjusted mean for Control toothpaste. ^c^Raw mean (standard error). ^d^Adjusted mean change from baseline (confidence interval) p-value [percentage change from baseline]. ^e^Supportive non-parametric analysis; p-value from van Elteren test.

### Tactile Threshold

**Fig 3:**
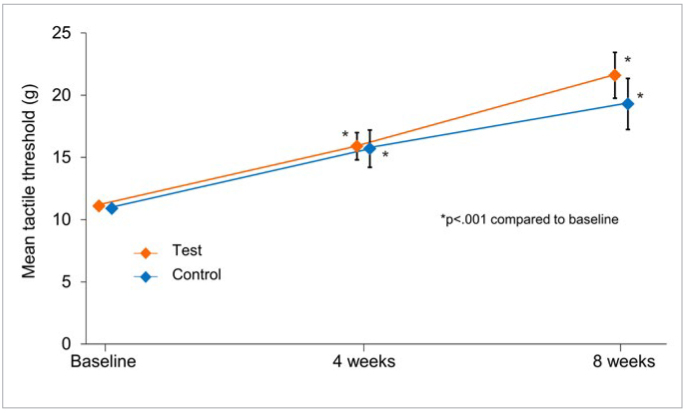
Tactile threshold by treatment group and visit (modified intent-to-treat population). Data shown are raw means ± standard errors and are offset for clarity; a high value is favourable. Tactile threshold range 0–80 g. Week 0 = baseline.

For tactile threshold, the results were not normally distributed; hence, the non-parametric Van Elteren test was used to assess effects of the treatments. For both study toothpaste groups, there was a decrease in DH, as shown by the statistically significant increase from baseline in tactile threshold at weeks 4 and 8 (p < 0.001 in all cases). There were no statistically significant differences between treatment groups (Fig 3; Table 2) with percentage differences of 0.0% at week 4 and 11.0% at week 8. Supporting non-parametric analysis inferred the same conclusions for between-treatment comparisons (Table 2).

### Safety

Study toothpastes were generally well tolerated. No treatment-emergent AEs were recorded.

## Discussion

Evaporative and tactile assessment techniques both attempt to measure DH; as such, the pattern of results in this study were anticipated to be similar. The primary endpoint, reduction in Schiff score at week 8, was statistically significantly greater with the SnF_2_ toothpaste compared with the Control. It was therefore surprising that there was little evidence of a between-treatment effect when DH was assessed using a tactile stimulus, since in other studies the improvements in Schiff sensitivity score were usually accompanied by improvements in tactile score.^7,8,26,27^

In previous DH studies of the experimental toothpaste formulation vs a control, effectiveness was shown from single to 14-day use with both tactile and evaporative stimuli.^7,8^ Closely related formulations have also shown superior performance by both assessment techniques in studies with identical design and duration to this study.^26,27^ Compared to the aforementioned studies, change from baseline in mean tactile threshold was considerably smaller for the Test toothpaste and considerably larger for the Control. Similar results were found in two China-based studies of anti-DH toothpastes, which used the same or similar study design/duration as here.^11,38^

There may be several reasons for the discrepancy between the two DH measures. DH studies, as they evaluate pain, are subjective and DH reductions may be manifestations of Hawthorne and/or placebo effects.^12,36^ There is considerable variation in degree and extent of DH exhibited from one person to another,^12^ in individual teeth in one person and in response to different stimuli, whether thermal, mechanical or chemical, in a single tooth.^25^ This favours the use of more than one assessment technique.^16^

The effect of ethnicity on pain perception was explored in a study that found distinct differences between Mandarin-speaking Chinese and ‘Western’ (Anglo-American, Swedish, Danish) dental patients.^23^ Chinese patients described pain caused by tooth drilling as dull and brief, whereas Western patients described it as sharp. Such differences may play a role in DH studies and might contribute to efficacy differences seen between this study and those previously conducted in US/UK participants.^26,27^ Further studies comparing responses to evaporative and tactile stimuli in Chinese and US/European populations may improve understanding of these results and methodologies.

The two endpoints were measured by separate examiners, although the same examiner performed all assessments for that endpoint. This is best practice to reduce the risk of bias, but increases the potential for inconsistent results between endpoints. However, the examiner who carried out the tactile assessment was trained and experienced in the technique and therefore we do not believe examiner to be a statistically significant factor.

No treatment-related AEs were recorded, which is consistent with previous long-term studies of SnF_2_ toothpastes in DH participants.^26,27,33,34^

## Conclusion

An experimental 0.454% SnF_2_ toothpaste demonstrated statistically significantly greater reductions in DH, as evaluated by Schiff sensitivity score after 4 and 8 weeks’ use, compared with a sodium monofluorophosphate-based Control toothpaste. However, there were no statistically significant differences between treatments when assessed by tactile threshold.
